# Proficient Novel Biomarkers Guide Early Detection of Acute Kidney Injury: A Review

**DOI:** 10.3390/diseases11010008

**Published:** 2022-12-30

**Authors:** Sahadeb Jana, Palash Mitra, Suchismita Roy

**Affiliations:** Nutrition Research Laboratory, Department of Paramedical Allied Health Sciences, Midnapore City College, Kuturiya, Bhadutala, Midnapore Pin-721129, India

**Keywords:** acute kidney injury, biomarkers, uromodulin, calprotectin, transferrin, nephrin

## Abstract

The definition of acute kidney injury (AKI), despite improvements in criteria, continues to be based on the level of serum creatinine and urinary output that do not specifically indicate tubular function or injury, or glomerular function or injury that is not significant enough to warrant acute hospitalization of the patient. Finding novel biomarkers of AKI has become a major focus nowadays in nephrology to overcome the further complications of end stage renal disease (ESRD). Many compounds, such as KIM 1, IL 18, NGAL, uromodulin, calprotectin, vanin 1, galactin 3, platelet-derived growth factor (PDGF), urinary Na^+^/H^+^ exchanger isoform 3 (NHE3), retinol binding protein (RBP) and Cystatin C, are released from the renal tubules and thus any alterations in tubular function can be detected by measuring these parameters in urine. Additionally, glomerular injury can be detected by measuring immunoglobulin G, nephrin, podocalyxin, podocin, transferrin, netrin-1, pyruvate kinase M2, etc. in urine. These novel biomarkers will be useful for timing the initial insult and assessing the duration of AKI. According to available research, these biomarkers could be applied to assess the onset of AKI, distinguishing between kidney injury and dysfunction, directing the management of AKI, and enhancing disease diagnosis. Therefore, we intend to present recent developments in our understanding of significant biomarkers implicated in various aspects of renal damage. Numerous biomarkers are implicated in various pathophysiological processes that follow renal injury, and can improve prognosis and risk classification.

## 1. Introduction

In clinical medicine, acute kidney damage (AKI) is a recurring problem. Despite substantial therapeutic advancements, AKI-related mortality and morbidity continue to be quite high. The absence of early indicators of renal failure is a significant contributor to this. The possibility of missing opportunities for therapeutic measures at a time while acute kidney injury may still be reversible or manageable arises from the AKI’s delayed identification. AKI causes approximately 2 million deaths worldwide each year [1] and is becoming more common in critically ill patients. The death rate for those who have the most severe form of the condition and need renal replacement treatment is between 50% and 80% [2]. AKI incidence is often reported as either community- or hospital-acquired AKI. AKI is typically acquired in hospitals in developed countries, but community-acquired AKI is more typical in low-income settings [3]. The Intensive Care Unit (ICU) is where it manifests itself most frequently and has a high death rate for the majority of older patients. Costs associated with AKI in these settings are substantially higher and more difficult to ignore. There is strong evidence that the occurrence of AKI is rising alarmingly, ranging from 5% of hospitalized patients to 30–50% of intensive care unit patients [4]. The morbidity and mortality related to AKI remain high despite major advancements in treatment approaches. Serum creatinine (sCr) and urine output are traditionally used as two functional biomarkers for the diagnosis of AKI, but these markers are insufficient since they show delayed responses after renal damage and have poor sensitivity and specificity. Creatinine does not rise until GFR or substantial parenchymal injury, and does not indicate a particular mechanism that causes GFR decline [5]. Whereas single or even combined biomarkers have yielded modest AKI differences, the practical use of the biomarkers investigated in AKI predictions continues to remain uncertain [6]. It has been demonstrated that several new biomarkers are more specific than sCr and can detect AKI early. Patients at high risk must be identified before renal insults cause kidney injury, and AKI must be diagnosed as soon as feasible, for any prophylactic methods to be effective. Early identification and prompt treatment of pharmacological characteristics that can induce AKI can lower morbidity and mortality because the timing of renal failure detection and mortality are directly correlated [7].

This article summarizes numerous unique genes and gene products that are regarded as novel biomarkers for diagnosing kidney disease at the very early stage as a result of the application of modern approaches such as molecular genome sequencing and proteomics to animal and human studies of AKI. This review article will discuss recent developments in knowledge of the most promising key biomarkers associated with various facets of renal injury.

## 2. Emerging Novel Biomarkers for Detecting Acute Kidney Injury

**Figure 1:**
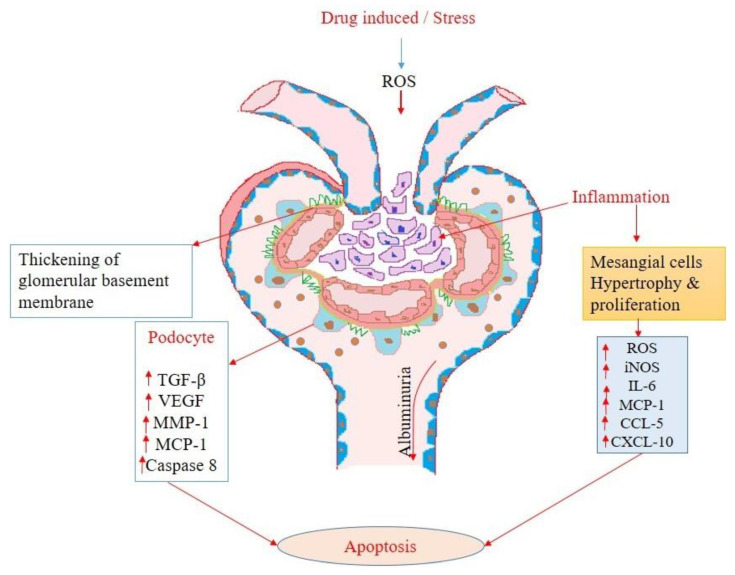
Glomerular structural alterations and damage results in release of several markers during the phases of acute kidney injury.

**Figure 2:**
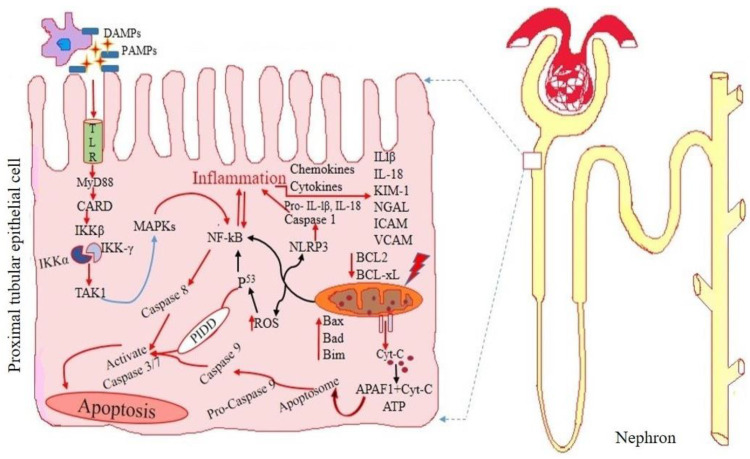
Drug-induced detailed mechanism of renal tubular apoptosis in acute kidney injury. Nephrotoxic drugs can cause increased oxidative stress by disrupting homeostasis mechanisms, increase free radical production and decrease the antioxidant defense system. This increases apoptosis and results in release of several biomarkers of tubular injury through urine during the early stage of kidney dysfunction.

It is necessary to identify patients with AKI early in order to provide timely treatment and enhance therapeutic outcomes. Detailed graphical representations of glomerular and tubular injury during acute kidney injury are shown in Figure 1 and Figure 2 accordingly.

### 2.1. Biomarkers for Glomerular Injury

#### 2.1.1. Immunoglobulin G

Immunoglobulin G (IgG) is a crucial globular antibody with 150 kDa that provides human immune protection against glomerular injury. The four human IgG subtypes differ structurally and are designed for a specific biological effect. During kidney disease the size of the glomerular capillary wall is significantly increased, which impairs the IgG and urine filtration from blood. On the other hand, IgG may be used as an indicator of glomerular membrane damage [8]. Numerous studies have reported severe pathological changes linked to higher urine levels and lower glomerular filtration rate (GFR) with IgG levels that severely impaired the renal function [9]. Another study reported that the IgG levels during urination significantly increased in diabetic patients and even in normoalbuminuria patients when compared to healthy controls. Consequently, IgG could be a more accurate biomarker for the early diagnosis of diabetic nephropathy than albuminuria [10]. Finally, IgG N-glycosylation sequences are related to a rapid loss in kidney function because IgG glycosylation is a critical post-translational event that plays a pathophysiological role in the development of diabetic nephropathy. IgG glycosylation is connected with the estimated GFR but not the albumin-to-creatinine ratio, meaning that these connections may indicate renal macroangiopathy rather than a microvascular infection [11]. Patients with idiopathic membranous nephropathy (IMN) may have urine IgG levels that indicate the severity of glomerular basement membrane damage. Urine IgG dramatically increased in IMN patients as proteinuria worsened. Additionally, 24-h albuminuria and ESR were positively connected with urine IgG, whereas albumin and eGFR were negatively correlated. The observation that urine IgG was strongly linked with 24-h urine protein levels, eGFR, and ESR, irrespective of other variables, lends more credibility to this. Hence, with proteinuria as a common diagnostic requirement for glomerular disorders and a standalone indicator of disease progression [12], IgG levels in the urine might indicate the level of an infection. The findings of this study support the idea that glomerular impairment rises with urine IgG production and may be connected to a higher risk of kidney diseases [13].

#### 2.1.2. Nephrin

Nephrin is a transmembrane protein with 180 kD that is produced in glomerular podocytes and is crucial for the development of the glomerular filtration barrier and maintenance of its effectiveness [14]. Irrespective of the condition, the primary function of nephrin is to stop proteins from passing through the glomerular barrier. Early cases of damaged podocytes include changes in the slit diaphragm, fusion of the filtration slit, apical displacement, remodeling of the foot process structure, and lastly, isolation from the basement glomerular membrane. Such alterations can cause severe and chronic glomerular damage that are found in a variety of glomerular infections: membranous glomerulonephritis, chrysanthemum nephropathy, collapse glomerulopathy, and localized segmental glomerulopathies are diseases that are only mildly invasive [14]. Nephrin can be seen in the urine due to any of these podocytopathies. Another study showed that urine nephrin is a more accurate diagnostic for the early diagnosis of diabetic nephropathy than albuminuria [15]. Nephrin production, phosphorylation, and down-regulation were changed by hyperglycemia, which also affected podocytes functionally and structurally. Nephronuria may precede microalbuminuria since it is present in all diabetic individuals with micro and macroalbuminuria and around 50% of those with normoalbuminuria. Additionally, an increase in the ratio of normoalbuminuria to macroalbuminuria results in a corresponding rise in urethral nephrin. It could highlight how podocyte metabolic activity plays a part in diabetes-related kidney disease [16].

#### 2.1.3. Podocalyxin

Podocalyxin (PCX) is a component of the CD34 family of proteins and is an anionic transmembrane sialoglycoprotein [17]. It is a crucial part of the slit diaphragm shape and is expressed on the apical side of podocyte foot processes. Therefore, the amount of podocalyxin in the urine may serve as a biomarker of podocyte dysfunction that shows the strength of the filtration barrier in the kidney [18]. Initial results investigating the early detection of PCX in the urine are currently being studied, despite the fact that the diagnostic potential of urinary PCX for chronic renal diseases is still not fully known. The findings revealed that individuals with diabetes mellitus had raised urinary levels of PCX, making it a more accurate and precise biomarker to be employed in the early identification of diabetic nephropathy compared to albuminuria [17]. In addition, the amount of urine PCX and the quantity of urinary podocytes was related to the percentage of segmental sclerosis in various glomerular disorders [19]. Correlations between urine PCX mRNA expression and commonly used renal function indicators were found (serum creatinine, eGFR, and albuminuria) [20].

#### 2.1.4. Podocin

The 43 kDa lattice protein podocin makes up the majority of the glomerular slit diaphragm. It is essential for podocyte structural and functional maintenance as well as nephrin-mediated cellular signaling [21]. A variety of renal diseases, including focal segmental glomerulosclerosis and renal insufficiency, are brought on by podocin mutations. The fast progression of renal dysfunction marked by mesangiosclerosis, glomerulonephritis, renal tubular damage, and nephropathy was demonstrated by investigation using mice as a model [22]. Studies revealed that podocin knockdown was associated with a range of glomerular injuries that showed a renal histopathological structure of damage dependent on the growth period. According to data from the literature, podocin may be an indicator for diabetic nephropathy that is linked to the intensity of the kidney dysfunction in the initial stages [23]. Despite the albumin to creatinine ratio, studies have demonstrated that podocin levels were greater in people who had diabetes than in a healthy control, including in those who had normoalbuminuria. On the other hand, GFR and serum albumin revealed a positive interaction with serum creatinine level and a negative association with urine podocin [23]. The presence of podocytes in the urine sediments, however, may be a strong indicator of serious damage and disease severity. When comparing the presence of urinary podocytes, they were found not to be present in diabetics with normoalbuminuria, diabetics having chronic renal disease or healthy controls, but were present in those with microalbuminuria and macroalbuminuria [24].

#### 2.1.5. Transferrin

Transferrin is a 79-kDa plasma protein that is made in the liver and released into the bloodstream where it binds to one or two Fe3^+^ atoms. Many different cell types then endocytose transferrin to transport Fe through certain plasma membrane receptors (TfR1 and TfR2) [25]. It is understood that transferrin is partially filtered across the glomerular filtration barrier and then quickly absorbed in the proximal tubular portion. TfRs and the megalin-cubilin compound, an additional transporter mechanism known to internalise numerous compounds containing transferrin, also are present in the luminal membrane of proximal tubule epithelial cells. A urinary biomarker of uranium-induced propensity to AKI was identified as transferrin. Gentamicin, cisplatin, and uranyl nitrate are examples of nephrotoxins known to accumulate in and harm the proximal tubules. Transferrin indicates the susceptibility brought on by these substances. Urinary transferrin is closely related to the degree of the renal dysfunction that results from exposure to a second insult that is not lethal to the rat models. Finally, the urine transferrin predicts in advance which oncological patients will show signs of nephrotoxicity under therapy with either cisplatin or carboplatin. In animal studies where risk was already induced by toxicants and drugs influencing the renal tubules (such as cisplatin, gentamicin, and uranyl nitrate), prior urinary transferrin levels correlate with the ensuing renal damage; in contrast, transferrin demonstrates no connection with the dangers posed by a drug influencing renal hemodynamics. In the danger phase (i.e., well before injury), transferrin accumulates mostly in urine specifically because osmosis is limited [26].

#### 2.1.6. Netrin-1

Netrin-1, a multifaceted neural regulatory protein, is highly expressed with in renal conditions. Recent studies showed various other functions of netrin beyond axonal guidance including development of mammary gland, pancreas, lung and blood vessels, inhibition of leukocyte migration during sepsis, mitogenesis and chemoattraction of endothelial cells. Although netrin-1 upregulation reduced ischemia-reperfusion-induced apoptosis and boosted tubular epithelial cell growth, netrin-1 deficiency in mice resulted in worsened AKI. In several AKI studies with animals in addition to data on humans with AKI caused by diverse factors (such as ischemic AKI, radio-contrast-induced AKI, septicemia AKI and including drug-induced AKI), it was shown that urine netrin-1 excretion rises exponentially. Studies show that just as quickly at 3 h after ischemia-reperfusion, netrin-1 is substantially increased in tubular epithelial cells, reaching a maximal level at 24 h. According to studies, netrin-1 may be detected in urine as soon as 1 h after various renal injuries. Additionally, examination of human urine samples with acute kidney injury revealed elevated netrin-1 levels throughout all categories of AKI [27]. A research study was conducted to look at how the expression of netrin-1 changed in individuals with severe AKI. Individuals with AKI who were infected had noticeably elevated urine netrin-1 concentration during the first hour. Healthy subjects’ urine samples did not show any variations in netrin-1 levels. Although serum creatinine concentration rises 24 h post ICU entrance, urine netrin-1 levels were increased as early as 1 h post ICU enrolment, suggesting that urinary netrin-1 is a helpful indicator for the initial identification of AKI in dialysis patients. Every individual indicator that reflects the many mechanisms that contribute to the pathogenesis of AKI is unlikely to be precise and strong, given the multifarious causes of AKI. Additional new biomarkers must be examined simultaneously in order to provide additional knowledge [28].

#### 2.1.7. Pyruvate Kinase M2

Pyruvate kinase M2 (PKM2), a glycolytic enzyme, has come to be recognized as a crucial enzyme that links infection and severe glycolysis. The enzyme pyruvate kinase, which catalyzes the concluding phase in glycolysis, has four isoforms: L, R, M1, and M2. PKM2 is one of these isoforms. In contrast to certain other isoforms, PKM2’s kinase activity includes the phosphorylation of proteins necessary for inflammation in addition to glycolysis. Through the regulation of cytokine production across transcription, PKM2 can also influence infection. According to a study, PKM2 suppression with shikonin reduces the effects of lipopolysaccharide-induced AKI by suppressing the production of (Hypoxia-inducible factor 1-alpha) HIF-1 and preventing apoptosis of proximal tubular epithelial cells [29]. Studies suggested that the predominant isoform of pyruvate kinase found in podocytes is PKM2. As podocytes are crucial in the etiology of AKI, PKM2 may provide an intriguing connection among metabolic and inflammatory changes during infection.

According to a study, chemotherapy administration dramatically elevated PKM2 activity in the glomerular cortex and medulla in an experimentally induced rat model of AKI. These results showed that chemotherapy treatment of human kidney (HK-2) cells boosted glycolysis and encouraged a metabolic switch from pyruvate to lactate for energy production. Researchers studying the metabolic state of renal tubular cells in both health and sickness are still learning about the unique roles which this enzyme plays in the juxtaglomerular apparatus, which is located near the renal cortex. Animals having podocyte-specific removal of PKM2 showed increased albuminuria and glomerular damage brought on by hyperglycemia. Although attempts have been made to describe PKM2’s activity in the kidneys, especially in the context of hyperglycemia-induced glomerular damage, more research has to be done on how it could play a role in the etiology of AKI [30].

### 2.2. Tubular Injury Diagnostic Markers

#### 2.2.1. Kidney Injury Molecule-1

KIM-1 is a type I transmembrane glycoprotein with a cell surface binding protein component and a molecular weight of 38.7 kDa. It is very marginally generated in the kidney as well as other tissues, but is markedly elevated in a variety of renal tubulo-interstitial diseases, polycystic kidney disease, and kidney damage, particularly following an ischemia-reperfusion event. In the urine of individuals exhibiting acute renal disease, soluble KIM-1 was found shortly after renal tubule injury. KIM-1 may be employed as an indicator for renal tubular damage, according to earlier investigations [31].

Increased urine KIM 1 levels are caused by the extracellular domain of KIM 1 being detached from the membrane in ischemic/toxic renal damage, a process that is contingent on a matrix-metalloproteinase (MMP). To fully understand the therapeutic consequences of KIM-1 in extracellular domain degradation, more study is necessary. By modifying translational modifications through the nuclear factor kappa-light-chain-enhancer of activated B-cells (NF-kB) pathway, KIM-1 inhibits proximal tubular cell cytokine release. Ongoing injury to different tubulointerstitial sections and the renal cortex is reflected in the expression of the KIM-1 gene in experimental settings. Increasing KIM-1 levels may be used to distinguish between CKD and acute kidney azotemia to determine acute tubular necrosis (ATN). Elevated KIM-1 levels have been proposed by a number of writers as a potential tool for identifying people at risk of developing AKI from CKD.

Furthermore, according to some publications, higher levels of KIM-1 in AKI patients may occur before histological alterations. The urine level of KIM-1 specifically for the prognosis of AKI was determined to be 86.0% and sensitivity to be 74.0% in a meta-analysis which included eleven trials and 2979 patients [32].

Recent research examined KIM-1’s potential for early diagnosis and/or prognostication of AKI onset in certain groups. NAG, NGAL, and urinary KIM-1 all showed potential for detecting renal damage following heart surgery. Increased levels were also found in diabetic nephropathy, and in transplant recipients; they may aid in the early identification of AKI caused by allograft rejection. KIM-1 is selectively elevated in dedifferentiated tubular cells after ischemia or nephrotoxic AKI, and is one of the most significantly induced proteins in the kidney following AKI in animal models. Urinary KIM-1 levels in hospitalized patients with established AKI predicted unfavorable clinical outcomes such the need for dialysis and mortality. It may be possible to distinguish between the expansion phase of AKI and recovery stages using urinary KIM-1 levels. It was discovered that IL-18 and KIM-1 together had the strongest predictive power for predicting severe AKI, and that urinary KIM-1 concentrations showed relatively low connection with other damage indicators.

KIM 1 can be used to identify renal dysfunction brought on by nephrotoxic medications because of its precision and accuracy in the early diagnosis of renal damage. Further research is necessary to verify novel biomarkers to be used in earlier detection, vulnerability assessments, and disease monitoring since AKI can have a number of etiologies, and its prevalence is rising. Organic cation transporter 2 (OCT2) and copper transporter 1 (CTR1) may be involved, as well as subsequent down-regulation of basolateral organic anion to renal transporter-mediated uptake [33]. Drug concentration in the proximal tubule is increased, which causes complexes to form and activate AMP protein kinase (AMPK). This causes reduction in autophagy, elevation in DNA damage, renal vascular resistance, necrosis of tubular cells, and levels of apoptosis and inflammation [34]. The primary site of cellular ATP generation and primary producer of ROS is the mitochondria. Stress on the cellular level impairs mitochondrial function by generating more ROS and less ATP. In addition to altering glutathione peroxidase (GPX) and superoxide dismutase (SOD), ROS increase the activity of nicotinamide oxidase 2 (NOX-2) and nicotinamide adenine dinucleotide phosphate (NADPH) [35]. ROS are overproduced as a result of DNA damage, which inhibits mitochondrial catalase (CAT), glutathione (GSH) and SOD, and increases the death of renal tubular cells. A crucial mechanism in the development of drug-associated AKI is inflammation. When tubular epithelial cells are injured, activated leukocytes and pro-inflammatory cytokines are needed, which start and extend the inflammation. The growth and progression of AKI are aided by activation of the NF-kB signaling system, which increases the release of TNF-, IL-1, IL-18, and KIM-1 by gene overexpression. Renal KIM-1 was inversely connected with renal function, positively associated with kidney damage, but not with albuminuria. Studies show that KIM-1 is connected with renal fibrosis and inflammation and is elevated in the very initial stages of kidney failure; as a result, urine KIM-1 may be employed as a quasi-initial biomarker in a variety of renal disorders [31].

#### 2.2.2. Interleukin 18

A member of the IL-1 superfamily, interleukin (IL)-18 is sometimes referred to as the interferon-gamma inducing factor. This cytokine promotes inflammation and the Th1 response. Both innate and adaptive immunity can be modulated by IL-18, and its instability can result in autoimmune or inflammatory disorders. After cleaving by caspase-1, IL-18 is released extracellularly as an 18 kDa bioactive mature molecule. It is first retained inside cells as a physiologically inactive 24 kDa progenitor [36]. IL-18 is produced by epithelial cells in the kidneys and is therefore tilized as a biomarker [37] and may help distinguish acute tubular necrosis from other etiological components of renal illness. IL-18 is upregulated in chronic renal disease and may play a role in the development of fibrosis. Increased levels of IL-18 have been linked to diabetic nephropathy, and they have been shown to be indicators of disease progression, the severity of albuminuria, and deterioration of kidney function [38]. IL-18 controls inflammation in a variety of ways. Clinical research has shown excellent results in a variety of situations where IL-18 is connected to an elevated inflammatory infiltration and more severe kidney injury. Studies have shown that IL-18 may be a key component in the pathogenesis of inflammatory kidney disorders, raising the possibility that IL-18 might be used in targeted therapy. Clinical research on IL-18 in inflammatory renal disease is lacking, nevertheless. Additionally, IL-18 function and signaling in inflammatory renal disease are not entirely understood. It is still unclear if IL-18 is definitively connected to disease pathogenicity. In experimental animal models, IL-18 signaling is regulated by IL-18 deficit, anti-IL-18 antibodies, IL-18R deficiency, and IL-18BP, all of which frequently have kidney-protective effects. Some contradictory findings imply that the corresponding signaling pathways, cytokine effects, etc. may not be in the same direction. Determining the pathophysiology of inflammatory kidney disease and developing treatments depend on investigations that would clarify IL-18 signalling [36].

#### 2.2.3. Neutrophil Gelatinase-Associated Lipocalin (NGAL)

A pervasive 21–25 kD iron-carrying protein of the lipocalin subfamily, neutrophil gelatinase-associated lipocalin (NGAL) is also known as siderocalin, lipocalin-2 (LCN2), or lipocalin. It is ubiquitously expressed within proximal tubules of the loop of Henle and collecting ducts [39]. It is released from renal tubular cells under any stress condition as well as from neutrophils during inflammation. It has also been found to have a role in kidney development and tubular regeneration after injury. High urinary NGAL can be used to predict AKI, discriminate intrinsic AKI from pre-renal AKI, predict renal non-recovery, in-hospital mortality, long-term CKD progression, ESRD, and mortality [40]. In AKI, in-hospital renal replacement therapy (RRT), or mortality prediction models formerly used urine NGAL levels, as it was discovered in clinical experiments to be strongly generated in response to tubular damage. Urine NGAL has been discovered to be an advance indicator of acute renal damage in later clinical investigations (AKI). After ischemia or nephrotoxic AKI in animal models, significantly upregulated proteins are found in the kidney. Its levels can be seen in patient plasma as quickly as 2 h after injury, reach their highpoint at around 6 h, and then remain high for up to five days before starting to decline [41].

Due to injury to the renal tubules, there is loss of NGAL into the urine, which has a strong association with estimated GFR, cystatin C, and serum creatinine. The extent of kidney damage is correlated with amount of NGAL expression, which may assist in distinguishing people who are more likely to show a quicker downturn in renal function. Renal injury, cytogenesis, and the development of CKD are all accelerated by NGAL activation [42]. Additionally, urine NGAL is a useful measure of normoalbuminuric renal illness in type 2 diabetes mellitus and a reliable indicator of renal damage prior to observable changes in eGFR [43].

#### 2.2.4. Uromodulin

The most prevalent protein in human urine is a glycoprotein called uromodulin, which is only generated in the kidneys, mostly by the endothelial cells of the thick ascending limb of the loop of Henle [44]. It can be a helpful biomarker for keeping an eye on kidney function, according to earlier research. There is a positive correlation between serum and urine uromodulin levels and glomerular filtration rate (eGFR). A protein redistribution from the epithelial cells of the thick ascending limb of the loop of Henle towards to the basolateral membrane and glomerular interstitial fluid was also demonstrated by molecular studies to occur within 48 h of an in vivo experiment using the hypoperfusion mechanism (IRI) of the kidneys. Uromodulin translocation may result in a rise in serum concentration, which might likely be employed as a prognostic indicator for gauging AKI recovery. Uromodulin concentrations were found to be lower in AKI patients than in healthy participants in recent investigations, and these low levels were linked to more severe stages of AKI. Uromodulin levels were separately linked with ESRD or quick loss of estimated GFR, and they were favorably correlated with estimated GFR and negatively correlated with proteinuria. It is highly likely that uromodulin has a beneficial influence in renal injury conditions because it effectively inhibits renal interstitium inflammation, lessens renal tubule damage, and speeds up the recuperation process [45].

#### 2.2.5. Calprotectin

A 24-kDa heterodimer called calprotectin is made up of the monomers S100A8 (10,835 Da) and S100A9, which bind calcium (13,242 Da) [46]. Neutrophils and monocytes, to a lesser extent, are its main sources. When originally discovered in the cytoplasm of neutrophil granulocytes, it was identified as an infectious protein. Moreover, renal epithelial cells and urine both contained the monomers S100A8 and S100A9 [47]. When released by activation of the immune cells, calprotectin serves as a danger-associated molecular sequence protein in addition to its primary role in cytoskeleton association. This calprotectin signal transduction is not specific to any one receptor; however, S100A8 and S100A9 have been identified as endogenous Toll-like receptor 4 activators. In relation to AKI, renal epithelial cells of the collecting duct were found to produce S100A8 and S100A9 in response to unilateral ureteral blockage. S100A8 and S100A9 are also specifically elevated in mice following ischemia reperfusion damage when invading renal neutrophils were the main source of S100A8/9. When there is early renal damage to wild-type mice, S100A9-knockout animals, who lack active calprotectin, exhibit greater fibrosis in renal tissues in response to hypoperfusion insult [48].

Innate immunity is also stimulated by calprotectin. When an invasive pathogen stimulates a neutrophil granulocyte, it subsequently releases prepared “damage-associated molecular pattern proteins”. One of these damage-associated molecular pattern proteins is calprotectin. It stimulates inflammatory processes by activating the Toll-like receptor 4 (TLR4) [49]. Prerenal AKI is the urinary equivalent of irritable bowel syndrome, according to studies. Despite viable epithelium structures in both circumstances, there is a functional impairment that results in very low levels of calprotectin concentrations. Numerous inflammatory renal disorders, such as vasculitis, glomerulonephritis and interstitial nephritis, are influenced by TLR4. TLR4 is activated in the collecting ducts, the thin limb of the loop of Henle, and the proximal and distal tubules in acute tubular necrosis (ATN), due to damage to the renal tubules. In these regions, TLR4 activity is induced in ischemia reperfusion damage [50]. Therefore, calprotectin could indicate all of the primary intrinsic AKI causes.

AKI outcome is largely dependent on early diagnosis and prompt treatment intervention. When the root cause of AKI is discovered, more targeted and personalized treatment options become available [51]. AKI can have a variety of causes, which are often categorized as prerenal, intrinsic (intrarenal), and postrenal. There are currently no viable indicators for the early identification of prerenal AKI. The response to fluid replacement is still regarded as the gold standard for separating prerenal from intrinsic AKI. While chronic renal failure suggests intrinsic AKI, returning kidney functions to normal by 24 to 72 h is thought to specify prerenal AKI. Calprotectin levels in the urine might be used as a technique to distinguish between intrinsic and prerenal AKI. When diagnosing intrinsic AKI, urine calprotectin levels were more accurate than other diagnostic indicators including proteinuria or fractional sodium excretion (FENa).

Ebbing et al. looked at changes in calprotectin levels over time in patients having already received surgery for kidney tumors, that causes iatrogenic renal ischemia reperfusion damage by temporary renal artery plugging. Urine calprotectin levels began to rise dramatically at the conclusion of surgery (about 2 h after ischemia), and they peaked 48 h later with a 69-fold increase above baseline values [52]. Studies suggest that calprotectin has a high degree of precision in discriminating between prerenal and intrinsic AKI. The ability of calprotectin to distinguish between prerenal and intrinsic acute kidney damage was investigated in a multicenter research. Particularly, individuals with intrinsic AKI had urine calprotectin levels that were 36 times higher than those with prerenal allograft kidney damage [53].

#### 2.2.6. Vanin 1

An ectoenzyme having pantetheinase activity known as vascular non-inflammatory molecule-1 (vanin 1) is glycosylphosphatidylinositol-anchored [54]. It has several significant qualities, including recycling of vitamin B5, which is crucial for the creation of coenzyme A, for energy production, as well as for inflammation and oxidative stress. Vanin 1 is mostly expressed in kidneys, liver, and gut [55]. In a previous study, it was shown that urine vanin 1 can serve as an early prognostic biomarker for renal tubular injury in normotensive rats with a high salt consumption. It was also discovered that urinary vanin 1 was detectable in experimental rats at the preliminary phase of hypertensive AKI. It was shown that in rats with nephrotoxicant-induced tubular damage to the kidneys, the urine vanin-1 concentration increased before the traditional indicators altered [56]. Depending on the precise etiology of the damage and any potential associations with illnesses, kidney expression of vanin 1 is controlled variably at both the gene and protein levels [55]. In investigations on humans, urine vanin 1 served as both an independent risk factor for renal function decrease in hypertension and an initial indicator of kidney damage linked with CKD [57]. Recent research discovered that streptozotocin-induced diabetic nephropathy in rats was associated with higher protein content of tubular vanin-1, and urine vanin-1 was found in diabetic nephropathy patients. Urinary vanin-1 is therefore considered to be a promising biomarker for the early recognition of AKI [54].

#### 2.2.7. Galectin-3

Galectin-3, a multifunctional member of the galactoside-binding lectin family with a range of biological functions, is a 32–35 kDa protein. Galectin-3 is widely distributed, which highlights the importance of this protein in a variety of processes, including cell attachment, cell proliferation and differentiation, embryogenesis, inflammation, cancer invasion, and metastasis. Galectin-3 has also been linked to cell fibrogenesis, and stiffness of the extracellular matrix. It is implicated in interactions between cells and the matrix [58]. Through processes independent of carbohydrates, it controls apoptosis and proliferation as well as the transport of proteins and glycolipids to the apical surface of epithelial cells [59]. In rats injected with or without nephrotoxic folic acid, animals with ischemia/reperfusion renal damage reported greater mRNA expression of galectin-3 at 2 h and high expression up to 48 h. The synthesis of galectin-3 mRNA and serum creatinine concentration were shown to be significantly negatively correlated. Galectin-3 expression was also restricted to proximal tubular cells before expanding towards a more distal part upon reperfusion. An important role of galectin-3 was found in the etiology of chronic kidney disease in addition to acute renal damage (CKD). A significant association between galectin-3 concentration in plasma and indicators of renal impairment, such as cystatin C levels, was observed in a research study including 133 patients with chronic heart failure. However after adjusting for floating indicators known to boost CKD prognosis as well as established clinical CKD determinants, higher blood galectin-3 levels were linked to an elevated risk of CKD. Galectin-3 showed a comparable, significant connection throughout a 10-year follow-up period. There was no correlation between the serum galectin-3 level and the albuminuria concentration [60]. In individuals experiencing renal failure, a rise in galectin-3 concentration is linked to kidney fibrosis, elevated chances of fast renal function decrease, the occurrence of chronic kidney disease, advanced renal impairment, and death [61]. According to the findings of a current study, patients with type 2 diabetes mellitus who had macroalbuminuria had considerably greater concentrations of galectin-3 than those who had renal function stage 4 and 5 CKD [62].

#### 2.2.8. Platelet Derived Growth Factor (PDGF)

The four polypeptide chains that make up the platelet-derived growth factor (PDGF) family are encoded by the PDGF-A, PDGF-B, PDGF-C, and PDGF-D genes, which are found on chromosomes 7, 22, 4, and 11, respectively [63]. PDGFs have specific binding affinities for the PDGF receptors (PDGFR-αα, -αβ, and -ββ). Injured epithelium cells release PDGFs during AKI, and mesangial cells, pericytes, and fibroblasts are among the cells that secrete PDGFs as CKD progresses [64]. PDGFR αα, PDGFR ββ, and PDGFR αβ are homodimers and heterodimers formed when the PDGF receptor (PDGFR) chain dimerizes. PDGFR α and PDGFR β forms were investigated in experimental kidney damage models [65]. PDGFRs are generally expressed on mesenchymal cells in the glomerulus and interstitium, whereas PDGFR α is principally exhibited on messangial cells, glomerular parietal, epithelial, and interstitial cells. However, PDGFR β is also widely expressed by kidney interstitial cells and to some extent by messangial cells. Matured podocytes and epithelial cells of the renal tubules, such as those in the collecting duct and urothelium, normally express the PDGFA chain in relation to PDGF ligands, while mesangial cells may do so in a negligible way. Prenatal death occurs in both PDGF-A and PDGF-B deficient animals. PDGF B-deficient animals have severely faulty glomerular development because of the lack of mesangial cells, despite the fact that PDGF A-deficient animals do not exhibit kidney related problems. Tyrosine kinase activation is required for PDGFR signaling. Tissue plasminogen stimulator, urokinase type activator, and plasmin are all capable of effecting the proteolytic activation of PDGF C and PDGF D that is necessary for receptor binding and activation. It is interesting to note that TGF-β and hepatocyte growth factor (HGF) levels are raised in the renal tubular fluid with glomerular albuminuria, and these factors may be directly responsible for elevating PDGF B expression in proximal tubulars, that would lead to interstitial fibrosis [66]. The S3 segments of the renal tubules express PDGF-B/PDGFR during the initial stages of ischemia reperfusion damage. This has to do with Src kinase-activated proliferation, which causes tubular epithelial cells to regenerate themselves. Parallel to these changes in renal hemodynamic, PDGF-B signaling plays a significant role in fibroblast transformation, capillary damage, and rarefaction. This suggests that PDGF plays a part in the development of the AKI-CKD transition [67].

#### 2.2.9. Urinary Na^+^/H^+^ Exchanger Isoform 3 (NHE3)

The most prevalent sodium transporter in renal tubules, the Na^+^/H^+^ exchanger isoform 3 (NHE3), an 85-kDa protein encoded by the SLC9A3 gene, is found in the apical membrane and subapical endosomes of renal tubular cells as well as in the apical membrane of loop of Henle. The reabsorption of 60% to 70% of filtered sodium is accomplished by NHE3. When reabsorption of sodium in the renal tubules is markedly reduced due to severe tubular injury, the NHE3 protein may be a useful marker to distinguish acute tubular necrosis (ATN) from many other renal diseases. NHE3 may be a more precise and sensitive biomarker of proximal tubular damage than other urine biological markers. There was a strong association among creatinine levels and urinary NHE3 levels, suggesting that the quantity of NHE3 protein present in the urine may also be a good indicator of the degree of tubular cell injury. Acute renal failure in humans may have a unique, accurate, and precise marker of renal tubular injury, according to studies on the urine NHE3 protein. Other than ATN, urinary NHE3 protein may be helpful in differentiating between ATN, prerenal azotemia, and intrinsic ARF [68]. Angiotensin II mediated hypertension and spontaneously hypertensive rats have long been linked to higher NHE3 activity and expression in the proximal tubules. NHE3 expression is elevated and Na+ reabsorption is increased in the renal tubules when oxidative stress levels are elevated. In the proximal tubules of rats used as a model for essential hypertension in humans, enhanced NHE3 expression or activity has been documented. Low nanomole concentrations of angiotensin II promote the production of NHE3 in cultivated proximal tubular cells, according to in vitro investigations. The expression of NHE3 and proximal Na+ reabsorption are both markedly elevated by low subpressor doses of angiotensin II, which aids in the progression of angiotensin II-dependent hypertension. However, in the renal tubular areas of the kidneys, NHE3 and angiotensin II have a direct causal link [69].

#### 2.2.10. Retinol Binding Protein

The transfer of vitamin A from the liver to other tissues is carried out by retinol binding protein (RBP), a small 21-kDa plasma protein that is also generated in the liver and has an affinity for particular small lipophilic molecules. The glomerulus freely filters it, and the proximal tubular portion afterwards reabsorbs and catabolizes it. According to a study, urine RBP levels are a highly sensitive predictor of renal tubule failure in people with AKI from a variety of causes [70]. According to studies, RBP and B2M levels are highly associated when urine pH is more than 6.0. As urine pH decreased, RBP/B2M ratios gradually increased, demonstrating RBP’s persistence in acidic urine as opposed to 2M’s volatility. Another study contends that elevated RBP levels in the initial two days of life have been strong predictors of clinically significant AKI and subsequent birth asphyxia in infants, a situation where the assessment of serum creatinine is particularly challenging because it significantly reflects maternal serum concentrations. In individuals with cisplatin, cadmium, mercury, lead, and cyclosporine induced nephrotoxicity, an elevated urinary RBP level has been found as an initial diagnostic biomarker of kidney dysfunction [71]. Urinary levels could conceivably produce a false negative outcome in this situation since plasma RBP levels are impaired in vitamin A insufficiency. Due to concurrent substantial glomerular albuminuria or hyperfiltration, conditions in which the renal tubular reabsorptive systems may be saturated, the usefulness of low-molecular-weight filtered proteins like RBP as markers in the context of AKI is constrained [72]. Urinary RBP levels can be a valuable indicator of the severity of the disease and the dynamics of its development or remission because they directly reflect the extent of renal tubular dysfunction. When the proximal tubules are unable to retain these low-molecular-weight proteins, RBP is present in urine at extraordinarily high concentrations and is extracted with the greatest intensity (>104-fold above “normal range”). Only in cases when the rate of glomerular filtration is normal or only slightly compromised does the expression pattern of urinary RBP reflect the renal tubules’ ability to absorb substances.

#### 2.2.11. Liver-Type Fatty Acid Binding Protein (L-FABP)

L-FABP, a 14 kDa protein, participates in intracellular fatty acid homeostasis in a variety of tissues and organs, including liver, mammalian intestinal mucosa, myocardium, adipose tissue, kidney, muscle, and other tissues. It binds to long chain fatty acids (LCFAs) and shifts them into mitochondria or peroxisomes, where the fatty acids became oxidized [73]. There have been numerous novel kidney damage biomarkers studied in both clinical and experimental settings. One of the most promising early indicators of AKI is urinary L-FABP. Both healthy and sick human kidneys express L-FABP, which was seen in the straight and convoluted section of the proximal tubules [74]. Free fatty acids are quickly converted to nonoxidized fatty acids, and so this conversion becomes more pronounced under various stressors, such as toxicity and ischemia, in addition to proteinuria [75]. Inducing the production of chemoattractants in the renal tubular region, L-FABP binds nonoxidized fatty acids strongly and causes tubulointerstitial damage. L-FABP excretion in the urine may be a valuable clinical diagnostic for identifying acute injury in the renal tubules is connected to the level of renal ischemia. It may also represent different types of stressors that can result in tubulointerstitial damage [76]. Before receiving coronary intervention or undergoing coronary angiography, patients with contrast induced-AKI may experience oxidative stress in the proximal tubules, which may cause fatty acid storage and the production of nonoxidized fatty acids within the cytoplasm (contrast-induced AKI) [77].

**Figure 3:**
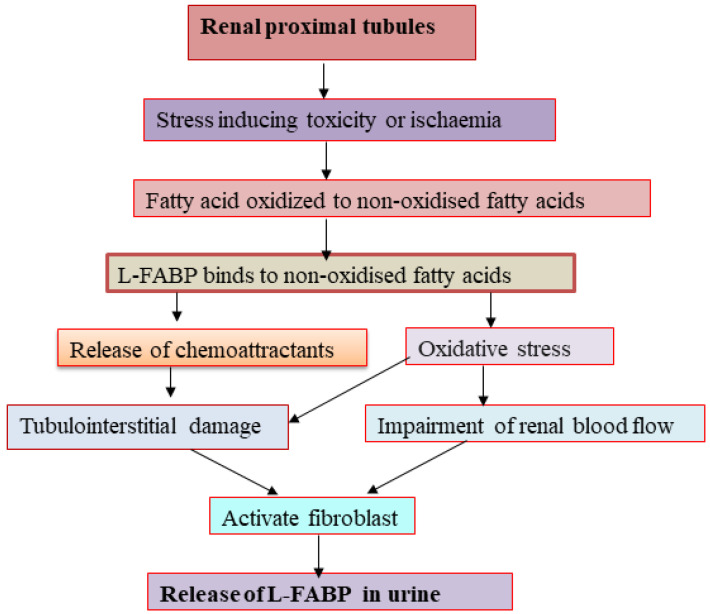
Mechanism of acute kidney injury related L-FABP release through urine at the time of tubular injury.

Chronic hypoxia in the kidney can result from structural changes that affect blood flow delivery, raise oxidative stress to the tubules, activate fibroblasts, and alter how the resident renal cells use their extracellular matrix. One aspect that needs to be taken into account as a pathway in urinary L-FABP excretion is chronic peritubular ischemia. Under tubular stress, proximal tubular cell expression of urinary L-FABP may be increased, leading to an increase in renal excretion of L-FABP out from proximal tubules prior to the onset of cellular damage in kidneys that develop AKI [76,78]. A graphical representation showing the release of L-FABP in the urine during AKI in Figure 3.

#### 2.2.12. β2-Microglobulin (B2M)

The human immune system contains the 11.8-kDa protein known as beta 2-microglobulin (also known as B2M), which is located just on the surface of nucleated cells. B2M travels through the glomeruli within kidneys before being reabsorbed by the renal proximal tubular area. B2M is typically only found in trace amounts in urine; however, when the renal tubules are injured, B2M concentrations rise as a result of the tubules’ diminished capacity to reabsorb this protein, acting as a functional indicator of tubular fibrosis. Increased filtration loads, such as those associated with chronic inflammatory disorders, or impaired glomerular filtration performance can also contribute to elevated serum B2M levels. Consequently, B2M may be a helpful biomarker to assess glomerular and tubular functioning. According to earlier research, SCr and the degree of tubulointerstitial lesions were strongly linked with urine B2M in individuals with IgA nephropathy. High levels of B2M are linked to both mortality and morbidity in end-stage renal disease (ESRD), including vascular calcification. Additionally, B2M is inversely correlated with glomerular filtration rate and positively correlated with inflammation and malnutrition [79]. Independent of muscle mass, serum B2M levels begin to increase early in renal failure. These characteristics have made blood B2M a suitable marker for evaluating renal function in AKI and CKD. Urinary B2M levels rise early during the course of nephrotoxicity mediated AKI in rat models and revert to normal during the healing phase [80].

#### 2.2.13. Cysteine-Rich Protein 61 (CYR61)

The extracellular matrix molecule cysteine-rich protein (CYR61) has four different domains. The physiological processes of angiogenesis, fibrosis, apoptosis, migration proliferation, differentiation, development and senescence are all impacted by the production of CYR61 [81]. Wound healing, and chronic inflammation harm to the tissue are all associated with high levels of CYR61. According to research, CYR61 overexpression may result from a reaction to environmental stress and/or hypoxia circumstances. Blocking CYR61, according to studies on mice with unilateral kidney ischemia reperfusion damage (IRI), may lessen the inflammatory effects of ischemic AKI. Clinical research using an animal model has shown that damaged kidney tissues express CYR61 differently. In animal models of obstructive kidney fibrosis and ischemia-reperfusion kidney damage, CYR61 expression was increased, which mediates proinflammatory effects. Whether the result is consequential or causal, a higher level of urine CYR61 implies active intrarenal inflammation and declining kidney function [82]. After induction of renal ischemia in rats, CYR61 is mainly activated in the proximal tubular part of the kidney within 3 to 6 h. It has been proposed as a potential diagnostic indicator for AKI in clinical and preclinical investigations since it is also released in the urine within 3 to 6 h [83]. One day after kidney damage, CYR61 could be found in the urine. Clinical research using an animal model has shown that kidney damaged tissues express CYR61 differently. In animal models of obstructive kidney fibrosis and ischemia-reperfusion kidney damage, CYR61 expression was increased, which mediates proinflammatory effects. Whether the result is consequential or causal, a higher level of urine CYR61 implies active intrarenal inflammation and declining kidney function [82]. One day after kidney damage, CYR61 could be found in the urine [84].

#### 2.2.14. Transforming Growth Factor Beta (TGF-β)

Three ubiquitous cytokines (numbered 1–3) that make up the TGF-β superfamily are collectively acclaimed as transforming growth factor beta (TGF-β). TGF-β is the most prevalent kind in mammals. A crucial regulatory phase of TGF-β activity, in addition to transcription, is stimulation from its reservoir, a dormant protein complex inside the extracellular matrix (ECM). TGF-β receptor activation results in intracellular signals that control a variety of physiological, pathological, and developmental processes, include response to chronic kidney injury [85]. Epidermal growth factor receptor (EGFR) and p53 are transactivated by TGF-β via ROS and proto-oncogene tyrosine protein kinase Src (c-Src)-dependent processes, which might result in renal fibrosis [86,87]. TGF-β1 signalling causes renal fibrosis and an increase in collagen production [88]. Because miR-21-deficient TGF-(1)-transgenic mice exhibit continued increase in proteinuria and glomerular damage in streptozotocin treated diabetic mice through activation of ROS pathway, this suggests a variety of roles for miR-21 as a feedback suppressor of TGF-/Smad3 signalling, including protection against kidney disease [89]. Smad (Suppressor of Mothers Against Decapentaplegic) is a protein that plays a key function in the transmission of receptor signals to particular target genes in the nucleus. Members of the Smad family, e.g., Smad2 and Smad3, were the first identified substrates of the TGF-β receptor 1 (TbRI) kinase [90]. The microRNAs miR-21, miR-192, and miR-29, which are engaged in renal fibrosis, are regulated by TGF-/Smad3, which also has a role in the pathogenesis of fibrosis in renal tissues. Smad4 contributes to the promotion of renal fibrosis caused by Smad3 [91]. The susceptibility to acute kidney damage (AKI) in diabetic nephropathy is significantly increased by TGF-/Smad3. Smad3 may interact with p53 and increase p53 activity, which may be one of the processes behind the kidneys’ vulnerability to AKI in diabetes patients [92].

#### 2.2.15. N-Acetyl-β-Glucosaminidase (NAG)

The proximal tubular lysosomal enzyme N-acetyl-glucosaminidase (NAG) catalyzes hydrolysis of glycoprotein terminal glucose residues. The two primary isoenzymes of NAG, “A” (acidic) and “B” (basic), are mostly found within human kidneys [93]. Enzymes with molecular weights greater than 70 kDa are unable to properly filter urine. NAG is excreted in urine in higher concentrations, and one early indicator of tubular injury is the presence of tubular cell damage brought on by nephrotoxicants such as pharmaceuticals, cadmium, and renal diseases such as diabetes, obstructive uropathy, and hypertension. For the diagnosis of glomerular function in chronic renal disorders, urinary NAG is helpful [94]. The use of urine NAG levels to indicate injury in a particular tubular section may be limited by the low threshold for the production of tubular enzymes in respect to any tubular injury. Additionally, urinary NAG is blocked by urea, which may reduce its prognostic usefulness [95,96]. Diabetes causes a progressive increase in urine NAG levels, fasting plasma glucose levels, and hemoglobin A1c. Depending on renal glucose concentrations, NAG may be released more often in the urine whenever the proximal tubular cells are exposed to elevated urinary glucose [97]. Increased NAG levels have been observed after tubular damage from nephrotoxicant exposure [98].

#### 2.2.16. Osteopontin

Originally derived from bone, osteopontin (OPN) is a phosphorylated glycoprotein that is also synthesized in healthy kidneys in pelvic epithelium, distal tubule epithelial cells, the thick ascending limb of the loop of Henle, the collecting duct, and Bowman’s capsule [99]. Inflammation causes osteopontin to be widely expressed and increased. OPN has been found to be a unique biomarker for renal outcome, overall survival, and injury severity in critically ill patients having AKI who require renal replacement therapy. Because inflammatory cells are retained and IL-6 is released, OPN may act as an indicator and mediator of multi-organ failure. Experimental and clinical research indicate that OPN is able to send survival signals to tubular epithelial cells and that it can prevent these cells in humans from going into apoptosis. Local OPN expression increased in case of damage in renal tubules. It is well established that OPN also functions as a protection factor in kidneys and guards tubular epithelial cells against apoptosis, similar to preliminary research on the role of OPN in AKI. Since the anti-apoptotic signals are no longer necessary once tubular epithelial cells have recovered from the injury, the tubular expression of OPN declines, leading to lower levels of OPN in the blood [100]. Immunoglobulin A nephropathy develops in part due to OPN (IgAN). IgA deposition in the glomerular mesangial cells is this disorder’s main defining hallmark [101]. In regions of tubulointerstitial damage, tubular epithelial cells and interstitial infiltrating cells have elevated expression of the OPN and CD44 receptors (the CD44 antigen is a cell surface glycoprotein engaged in cell to cell interaction, migration, and cell adhesion). According to research, proteinuria and membranous glomerulonephritis (MGN), the second most common cause of progressive kidney disease in adults, are related to the presence of OPN in IgAN patients’ urine [102]. Recent research showed that the proximal tubules of people with both progressive and nonprogressive MGN overexpressed OPN [103]. In the AKI patients, there was a notable link between both the mRNA and protein expression of OPN. Increased macrophage infiltration as well as CD4 and CD8 infiltration were linked to high OPN expression in the kidneys. OPN causes NfB to activate, which might cause glomerular injury [104]. As a result, it was proposed that OPN might be an useful biomarker of MGN development. Patients with diabetic nephropathy have elevated levels of OPN in their glomeruli and tubular epithelial cells of the renal cortex [105].

#### 2.2.17. Clusterin

Heat shock protein clusterin, which has a molecular weight of 75–80 kDa, is produced during stress by both epithelial and secretory cells. In murine kidneys, it has been shown to play both anti-apoptotic and protective roles towards renal ischemia-reperfusion injury [106]. Furthermore, renal tubular epithelial and mesangial cells also contained clusterin. Its reduced expression made glomerulopathy-related proteinuria and postischemic renal damage worse. After ischemia-reperfusion damage, clusterin deficient mice experienced increasing renal inflammation and fibrosis [107]. Studies on humans are limited to the one study on urinary clusterin in diabetes mellitus patients and the encouraging outcomes of the testing multi-biomarker kit containing urinary clusterin in the range of indicators of acute and chronic renal damage. During ischemia/renal reperfusion, ureter obstruction, and toxicant-induced kidney injury (gentamicin, folic acid), clusterin mRNA is upregulated [108]. Both cells with a normal appearance and necrotic renal tubules have immunoreactive clusterin when there is a disease. It is possible that clusterin contributed to the early nonjunctional cell aggregation. As a result, junctional connections arise between cells and cell adhesion molecules are expressed. The mediation of morphologically differentiated nodules made up of non-proliferating cells buried in matrix by vascular smooth muscle cells cultured is one example of a more complex cell interaction that clusterin may also be involved in. When a cell is under stress, secreted clusterin, which is engaged in lipid transport, is released. By lowering oxidative stress and interacting with misfolded proteins, it protects the cell. Urinary clusterin produced in mouse kidney damage models has antifibrotic properties [109]. On the other hand, apoptosis upregulates nuclear clusterin, which then uses Bax to support apoptosis. The urogenital tract should be the local source of clusterin in urine as a result of glomerular size modification. When a medication causes kidney damage, the distal and proximal tubules, glomerulus, and collecting ducts activate and release the secreted isoform [110]. Increased urine clusterin may also be influenced by changes in renal tubular function because clusterin released to the proximal convoluted tubule is the ligand for megalin-facilitated endocytosis.

#### 2.2.18. IL-6

It has long been known that the pleiotropic cytokine IL-6 does have pro- and anti-inflammatory effects [111]. Endothelial cells produce a large amount of IL-6 in response to pro-inflammatory signals such as TNF- and hypoxia. It is also frequently produced in reaction to tissue damage and organ failure [112]. When tissues are injured, IL-6 is quickly generated at the initial stage of the inflammatory response. In response to infection and tissue damage, it encourages the development and activation of T-cells, B-cells, macrophages, etc. In patients who had stage 3 AKI compared to non-AKI patients, higher blood IL-6 levels were substantially associated with a higher risk of developing AKI. The acute-phase proteins CRP, fibrinogen, and serum amyloid A are all stimulated during the inflammatory immune response by IL-6, while albumin, fibronectin, and transferrin synthesis are suppressed. Strongly linked with kidney injury was IL-6 expression, which was greatly increased in kidney (113-fold), primarily in epithelial cells of renal tubules. A lack of IL-6 reduced neutrophil accumulation and made mice more resilient to the injury. Additionally, nephrotoxin-induced damage was significantly reduced in wild-type mice after neutrophil depletion. Thus, one of the main causes of AKI is neutrophil activation driven by IL-6. Contrary to popular belief, activation of the IL-6 trans-signaling pathway greatly reduces kidney damage while maintaining renal function [113]. The observation was made using an AKI model with ischemia-reperfusion injury.

#### 2.2.19. E-Cadherin

E-cadherin, also known as epithelial cadherin or uvomorulin, is a 120 kDa transmembrane glycoprotein that is a member of the traditional cadherin family. It is crucial for morphogenesis and wound healing, and it performs a calcium-dependent function in cell-cell adhesion. An adhesion protein called E-cadherin is released into the bloodstream as a result of cell-cell separation [114]. By controlling the actin cytoskeleton, E-cadherin, a key component of tubular adherent proteins, maintains intercellular connections and cell polarity in epithelial tissue. An extensive research on E cadherin was done in kidney fibrotic disorders [115]. Evidence, however, emphasized the role of E-cadherin in the epithelial-mesenchymal transition (EMT) in the development of kidney related disorders. Recent research has repeatedly demonstrated that cisplatin causes a decrease in E-cadherin levels during the acute phase of damage. The 80 kDa ectodermal component of the protein, which may be broken down by a number of proteases such as plasmin, MMP3, MMP7, ADAM10, and ADAM-15, correlates to e-cadherin as a soluble fragment in the urine [116]. A combination immunoprecipitation and MALDI-TOF mass spectrometry analysis was performed to determine if the E-cadherin was present in the urine of diabetic nephropathy (DN) patients that corresponded a 80 kDa fragment. The 80 kDa protein, which could be enhanced from the urine of DN patients with microalbuminuria, was clearly visible in the mass spectra. The 80 kDa ectodomain of E-cadherin is the same as the urine E-cadherin seen in patients with DN, pointing to a decline in the renal tubule caused by diabetes. According to research, diabetic people who are at risk of developing renal illness may benefit from using the E-cadherin as an early predictive biomarker. Additionally, it appears that the release of E-cadherin in diabetes patients’ urine is an early sign of impaired kidney function and epithelial injury [117].

#### 2.2.20. Calbindin

Calbindin-D28k is a widely distributed D-vitamin-dependent cytosolic calcium binding protein that is present in many different tissues and in many different species of mammals [118]. The primary cells of the collecting and distal convoluted tubules in the kidney, where calcium reabsorption is controlled, express calbindin. The expression of calbindin is altered by injury to the distal section of the nephron, which also modifies the concentration of calbindin in the urine. However, very little is known about the intrarenal control of calbindin release and expression during injury to the kidneys. Within two days of receiving cisplatin treatment, the quantities of the proteins calbindin and KIM 1 in the urine increased by 11.6 and 2.5 times, respectively. In mice treated with cisplatin, levels of blood urea nitrogen and serum creatinine increased by three days, confirming the progression of acute renal injury. Days 3 and 4 following cisplatin therapy showed time-dependent reductions in intrarenal calbindin, while day 3 saw a 200-fold elevation of calbindin (CALB1) and KIM-1 messenger RNAs (mRNAs) in the urine. According to research, early renal calbindin level reductions and calbindin protein release into the urine trigger a compensatory elevation of mRNA expression at later time periods. Calbindin’s utility as a strong urine biomarker of renal dysfunction is further enhanced by understanding how it is regulated during cisplatin nephrotoxicity. The combination use of a proximal (Kim-1) and distal tubular (calbindin) marker to detect acute kidney injury after cisplatin treatment has been supported by studies [119]. Surprisingly, the level of calbindin in the urine satisfies the requirements for usage as an optimal biomarker for the prognosis of kidney injury because it is only released from injured kidneys’ distal tubular cells, where it is engaged in Ca^2+^ reabsorption. Additionally, the distal convoluted tubular portion of nephron can be injured, and calbindin can be utilized to track the genesis and progression of this injury [120].

#### 2.2.21. Cystatin C

CysC is a 13-kDa endogenous cysteine proteinase inhibitor and a member of the protein family that is important for the breakdown of different peptides and proteins inside of cells. Every nucleated cell in the human body produces CysC, which is then released into the plasma at a rather steady pace [121]. CysC is created at a comparatively steady rate, released into the plasma, and 99% of it is filtered by glomeruli, making it a useful biomarker of impaired kidney function [122]. Regardless of muscle mass, all nucleated cells produce the endogenous biomarker of renal function known as cystatin C (CysC), which is then removed from circulation via glomerular filtration without reabsorption or secretion. In the majority of AKI cases, CysC performs equally well as Scr as a biomarker of renal function and even outperforms Scr in some instances. There is some evidence that CysC concentration rises before Scr does during AKI, suggesting that it may be able to identify renal impairment earlier than creatinine. CysC levels throughout the phase of renal recovery are less well understood. In the field of AKI research, accurately predicting renal function recovery after AKI is a long sought aim. Early AKI recovery detection may lead to less intensive resource use and faster hospital discharge. In CKD, serum CysC levels have a strong correlation with GFR measurements. They also clearly correlate with both all-cause and cardiovascular-specific mortality. Therefore, a decline in GFR and an increase in serum CysC level are closely related. Furthermore, CysC’s intra-individual variability seems equivalent to SCr’s. Recently developed and verified estimation formulas for GFR depend on serum CysC. In addition, uCysC has been demonstrated to be independently linked with death in severely ill patients with AKI. Elevation of sCysC predates small declines in GFR 1 to 2 days prior to symptoms, SCr elevation, and/or renal function decline. In some patient populations, particularly those with reduced serum concentration of creatinine such as older adults, young kids, patients with cirrhosis, renal transplant recipients and malnourished individuals, it has been suggested that CysC-based estimates of GFR may perform much better than creatinine-based estimates. CysC levels in people with diabetes without renal damage were observed to rise together with HbA1c levels. According to studies, serum CysC levels are linked to insulin resistance irrespective of renal function, although HbA1c levels have been shown to raise it independently. A growing body of research indicates that high HbA1c levels may interfere with serum CysC concentrations [123].

#### 2.2.22. TIMP-2 × IGFBP7

Two proteins that are secreted in the urine by renal tubular cells in response to injury are insulin-like growth factor binding protein 7 (IGFBP7) and urinary tissue inhibitor of metalloproteinases-2 (TIMP-2). These compounds are secreted when tubular cells are inflamed or are ischemic in order to stop the cell cycle of nearby renal tubules [124]. The association between elevated urine TIMP2 and IGFBP7 concentrations and the probability of developing AKI has been established by numerous investigations. In the presence of hypoperfusion and hypoxia, ROS may induce the upregulation of both of these biomarkers. Potential stress insults that result from ROS generation trigger the production of TIMP-2 and IGFBP7. The effectiveness of the TIMP-2and IGFBP7 in the early diagnosis and risk stratification of AKI has been assessed in a number of clinical investigations [125]. Measurements of TIMP-2 and IGFBP7 at ICU admission immediately following cardiac surgery predict AKI within 48 h after surgery, independently of clinical variables such as preoperative renal function, and with greater specificity. Using the cell cycle arrest markers TIMP-2 and IGFBP7, a study was conducted and the emergence of moderate-to-severe AKI in critically sick, postoperative surgical patients was predicted. The concept of AKI as secondary damage happening as danger- and pathogen-associated molecular pattern (DAMP, PAMP) molecules are delivered to the tubule via both glomerular filtration and the circulation is the direction of current mechanistic thought in AKI. These substances signal a variety of cell responses, including changes in cell cycle progression, when they are recognized by pattern recognition receptors on the tubular cell surface. Cell cycle arrest can result in senescence and/or apoptosis if it lasts too long. Cell cycle arrest, on the other hand, is a defense mechanism that stops dividing cells when they might be harmed. These biomarkers appear to be resilient to the type and urgency of surgery, and the fact that they work so effectively in surgical patients shows that this pathway of AKI is regulated much more by the host’s reaction to stress than by the actual sort of operation. The G1 cell cycle arrest phase, which is observed to occur at the very early phases of cellular stress, has been linked to both TIMP2 and IGFBP7 [126]. In particular, it has been demonstrated that a number of insults can lead renal tubular cells to enter this G1 cell cycle arrest stage after being stressed. Due to their roles in cell cycle arrest and signal transduction in innate immune activation, TIMP2 and IGFBP7 appear to function more effectively in surgical patients than previously reported biomarkers [127]. Concentrations of TIMP2 and IGFBP7 seem to be a separate component linked to the onset of severe AKI. Early indicators of tubular damage are urinary TIMP2 and IGFBP7 values, which also serve as short-term predictors of severe AKI. On the other hand, urine production and serum creatinine are recognized as late indicators of renal function. The onset of renal dysfunction would be linked to the early tubular injury identified by the release of TIMP2 and IGFBP7. Kidney function after 24 h appears to be poorly correlated with TIMP2 and IGFBP7. TIMP2 and IGFBP7 should only be viewed as a momentary representation of renal health [128].

## 3. Conclusions

Several different biomarkers were listed in this review article. One hundred biomarkers were found in the relevant examination of the literature, some of which were carefully checked, but no biomarker has been introduced into routine clinical practices as an unrivalled biomarker. Owing to multiple aforementioned limitations, this situation will not change as yet. Although initial biomarker analysis has given an innovative approach to personalized results in AKI, it also carries significant limitations, especially in terms of costs. From a pre-clinical standpoint, the first objective is to utilize a good comparator to evaluate the use of positive biomarkers in the initial diagnosis of AKI. The first stage is to identify the community of persons at high risk to develop AKI based on a thorough and critical examination of patients. Additionally, it is probable that the AKI panels will aid in differentiating between the different kinds and etiologies of AKI in light of the variable expression of the biomarkers.

## Data Availability

MDPI Research Data Policies.

## References

[1] Kellum J.A., Lameire N., Aspelin P., Barsoum R.S., Burdmann E.A., Goldstein S.L. (2012). KDIGO AKI guidelines. Kidney Int..

[2] Lewington A.J., Cerdá J., Mehta R.L. (2013). Raising awareness of acute kidney injury: A global perspective of a silent killer. Kidney Int..

[3] Susantitaphong P., Cruz D.N., Cerda J., Abulfaraj M., Alqahtani F., Koulouridis I., Jaber B.L. (2013). World incidence of AKI: A meta-analysis. Clin. J. Am. Soc. Nephrol..

[4] Xue J.L., Daniels F., Star R.A., Kimmel P.L., Eggers P.W., Molitoris B.A., Himmelfarb J., Collins A.J. (2006). Incidence and Mortality of Acute Renal Failure in Medicare Beneficiaries, 1992 to 2001. J. Am. Soc. Nephrol..

[5] Parikh C.R., Thiessen-Philbrook H., Garg A.X., Kadiyala D., Shlipak M.G., Koyner J.L., Edelstein C.L., Devarajan P., Patel U.D., Zappitelli M. (2013). Performance of Kidney Injury Molecule-1 and Liver Fatty Acid-Binding Protein and Combined Biomarkers of AKI after Cardiac Surgery. Clin. J. Am. Soc. Nephrol..

[6] Malyszko J. (2010). Biomarkers of Acute Kidney Injury in Different Clinical Settings: A Time to Change the Paradigm?. Kidney Blood Press. Res..

[7] Zhang W.R., Parikh C.R. (2019). Biomarkers of Acute and Chronic Kidney Disease. Annu. Rev. Physiol..

[8] Hou J., Cheng Y., Hou Y., Wu H. (2019). Lower Serum and Higher Urine Immunoglobulin G Are Associated with an Increased Severity of Idiopathic Membranous Nephropathy. Ann. Clin. Lab. Sci..

[9] Hu Q., Wu K., Pan W., Zeng Y., Hu K., Chen D., Huang X., Zhang Q. (2020). Intestinal flora alterations in patients with early chronic kidney disease: A case-control study among the Han population in southwestern China. J. Int. Med Res..

[10] Abdou A.E., Anani H.A., Ibrahim H.F., Ebrahem E.E., Seliem N., Youssef E.M., Ghoraba N.M., Hassan A.S., Ramadan M.A.A., Mahmoud E. (2021). Urinary IgG, serum CX3CL1 and miRNA-152-3p: As predictors of nephropathy in Egyptian type 2 diabetic patients. Tissue Barriers.

[11] Singh S.S., Heijmans R., Meulen C.K., Lieverse A.G., Gornik O., Sijbrands E.J., Lauc G., van Hoek M. (2020). Association of the IgG N-glycome with the course of kidney function in type 2 diabetes. BMJ Open Diabetes Res. Care.

[12] Cravedi P., Remuzzi G. (2013). Pathophysiology of proteinuria and its value as an outcome measure in chronic kidney disease. Br. J. Clin. Pharmacol..

[13] Bazzi C., Rizza V., Casellato D., Tofik R., Berg A.-L., Gallieni M., D’Amico G., Bakoush O. (2014). Fractional excretion of IgG in idiopathic membranous nephropathy with nephrotic syndrome: A predictive marker of risk and drug responsiveness. BMC Nephrol..

[14] Kandasamy Y., Smith R., Lumbers E.R., Rudd D. (2014). Nephrin–a biomarker of early glomerular injury. Biomark. Res..

[15] Kostovska I., Tosheska-Trajkovska K., Topuzovska S., Cekovska S., Spasovski G., Kostovski O., Labudovic D. (2019). Urinary nephrin is earlier, more sensitive and specific marker of diabetic nephropathy than microalbuminuria. J. Med. Biochem..

[16] Silambanan S., Kondapi K., Kumar N.L., Moorthy S. (2021). A study of association of urinary nephrin with albuminuria in patients with diabetic nephropathy. Indian J. Nephrol..

[17] Kostovska I., Trajkovska K.T., Cekovska S., Topuzovska S., Kavrakova J.B., Spasovski G., Kostovski O., Labudovic D. (2020). Role of urinary podocalyxin in early diagnosis of diabetic nephropathy. Rom. J. Intern. Med..

[18] Akankwasa G., Jianhua L., Guixue C., Changjuan A., Xiaosong Q. (2018). Urine markers of podocyte dysfunction: A review of podocalyxin and nephrin in selected glomerular diseases. Biomark. Med..

[19] Asao R., Asanuma K., Kodama F., Akiba-Takagi M., Nagai-Hosoe Y., Seki T., Takeda Y., Ohsawa I., Mano S., Matsuoka K. (2012). Relationships between Levels of Urinary Podocalyxin, Number of Urinary Podocytes, and Histologic Injury in Adult Patients with IgA Nephropathy. Clin. J. Am. Soc. Nephrol..

[20] Zeng J., Zhang X., Yu R., Tang Y., Luo W.-J., Chen C., Wu Y.-J. (2015). Research on the Combined Detection of Urine UmAlb and Urinary Nephrin, Podocalyxin in Podocyte of MKR Mice with Diabetic Nephropathy. Sichuan Da Xue Xue Bao. Yi Xue Ban = J. Sichuan Univ. Med. Sci. Ed..

[21] Huber T.B., Simons M., Hartleben B., Sernetz L., Schmidts M., Gundlach E., Saleem M.A., Walz G., Benzing T. (2003). Molecular basis of the functional podocin–nephrin complex: Mutations in the NPHS2 gene disrupt nephrin targeting to lipid raft microdomains. Hum. Mol. Genet..

[22] Mollet G., Ratelade J., Boyer O., Muda A.O., Morisset L., Lavin T.A., Kitzis D., Dallman M., Bugeon L., Hubner N. (2009). Podocin Inactivation in Mature Kidneys Causes Focal Segmental Glomerulosclerosis and Nephrotic Syndrome. J. Am. Soc. Nephrol..

[23] Elshaarawy A., Behairy M.A., Bawady S.A., Abdelsattar H.A., Shadad E. (2018). Urinary podocin level as a predictor of diabetic kidney disease. J. Nephropathol..

[24] Abdel Rahman H.S., Hadhoud K., Bakr H.G., Youssef K.M. (2019). Assessment of urinary podocin level as an early indicator in diabetic nephropathy. Zagazig Univ. Med. J..

[25] Zhang D., Meyron-Holtz E., Rouault T.A. (2007). Renal Iron Metabolism: Transferrin Iron Delivery and the Role of Iron Regulatory Proteins. J. Am. Soc. Nephrol..

[26] Vicente-Vicente L., Ferreira L., González-Buitrago J.M., López-Hernández F.J., López-Novoa J.M., Morales A.I. (2013). Increased urinary excretion of albumin, hemopexin, transferrin and VDBP correlates with chronic sensitization to gentamicin nephrotoxicity in rats. Toxicology.

[27] Brian Reeves W., Kwon O., Ramesh G. (2008). Netrin-1 and kidney injury. II. Netrin-1 is an early biomarker of acute kidney injury. Am. J. Physiol.-Ren. Physiol..

[28] Tu Y., Wang H., Sun R., Ni Y., Ma L., Xv F., Hu X., Jiang L., Wu A., Chen X. (2014). Urinary netrin-1 and KIM-1 as early biomarkers for septic acute kidney injury. Ren. Fail..

[29] Wu J., Rong S., Zhou J., Yuan W. (2021). The role and mechanism of PKM2 in the development of LPS-induced acute kidney injury. Histol. Histopathol..

[30] Alquraishi M., Chahed S., Alani D., Puckett D.L., Dowker P.D., Hubbard K., Zhao Y., Kim J.Y., Nodit L., Fatima H. (2022). Podocyte specific deletion of PKM2 ameliorates LPS-induced podocyte injury through beta-catenin. Cell Commun. Signal..

[31] Geng J., Qiu Y., Qin Z., Su B. (2021). The value of kidney injury molecule 1 in predicting acute kidney injury in adult patients: A systematic review and Bayesian meta-analysis. J. Transl. Med..

[32] Medić B., Rovčanin B., Jovanović G.B., Radojević-Škodrić S., Prostran M. (2015). Kidney Injury Molecule-1 and Cardiovascular Diseases: From Basic Science to Clinical Practice. BioMed Res. Int..

[33] Estrela G.R., Wasinski F., Felizardo R.J.F., Souza L.L., Câmara N.O.S., Bader M., Araujo R.C. (2017). MATE-1 modulation by kinin B1 receptor enhances cisplatin efflux from renal cells. Mol. Cell. Biochem..

[34] Xu Y., Ma H., Shao J., Wu J., Zhou L., Zhang Z., Wang Y., Huang Z., Ren J., Liu S. (2015). A Role for Tubular Necroptosis in Cisplatin-Induced AKI. J. Am. Soc. Nephrol..

[35] Qi Z., Li Z., Li W., Liu Y., Wang C., Lin H., Liu J., Li P. (2018). Pseudoginsengenin DQ exhibits therapeutic effects in cisplatin-induced acute kidney injury via Sirt1/NF-κB and caspase signaling pathway without compromising its antitumor activity in mice. Molecules.

[36] Hirooka Y., Nozaki Y. (2021). Interleukin-18 in Inflammatory Kidney Disease. Front. Med..

[37] Parikh C.R., Devarajan P. (2008). New biomarkers of acute kidney injury. Crit. Care Med..

[38] Araki S., Haneda M., Koya D., Sugimoto T., Isshiki K., Chin-Kanasaki M., Uzu T., Kashiwagi A. (2007). Predictive impact of elevated serum level of IL-18 for early renal dysfunction in type 2 diabetes: An observational follow-up study. Diabetologia.

[39] Guo L., Zhu B., Yuan H., Zhao W. (2020). Evaluation of serum neutrophil gelatinase-associated lipocalin in older patients with chronic kidney disease. Aging Med..

[40] McMahon B.A., Galligan M., Redahan L., Martin T., Meaney E., Cotter E.J., Murphy N., Hannon C., Doran P., Marsh B. (2019). Biomarker Predictors of Adverse Acute Kidney Injury Outcomes in Critically Ill Patients: The Dublin Acute Biomarker Group Evaluation Study. Am. J. Nephrol..

[41] Soto K., Papoila A.L., Coelho S., Bennett M., Ma Q., Rodrigues B., Fidalgo P., Frade F., Devarajan P. (2013). Plasma NGAL for the Diagnosis of AKI in Patients Admitted from the Emergency Department Setting. Clin. J. Am. Soc. Nephrol..

[42] Rysz J., Gluba-Brzózka A., Franczyk B., Jabłonowski Z., Ciałkowska-Rysz A. (2017). Novel Biomarkers in the Diagnosis of Chronic Kidney Disease and the Prediction of Its Outcome. Int. J. Mol. Sci..

[43] Li A., Yi B., Liu Y., Wang J., Dai Q., Huang Y., Li Y.C., Zhang H. (2019). Urinary NGAL and RBP Are Biomarkers of Normoalbuminuric Renal Insufficiency in Type 2 Diabetes Mellitus. J. Immunol. Res..

[44] Lhotta K. (2010). Uromodulin and Chronic Kidney Disease. Kidney Blood Press. Res..

[45] El-Achkar T.M., McCracken R., Liu Y., Heitmeier M.R., Bourgeois S., Ryerse J., Wu X.-R. (2013). Tamm-Horsfall protein translocates to the basolateral domain of thick ascending limbs, interstitium, and circulation during recovery from acute kidney injury. Am. J. Physiol. Physiol..

[46] Stríz I., Trebichavský I. (2004). Calprotectin—A pleiotropic molecule in acute and chronic inflammation. Physiol. Res..

[47] Moghtaderi M., Vakili M., Fahimi D., Esfahani S.-T., Sharifzadeh M. (2021). Comparative analysis between urinary calprotectin and serum creatinine for early detection of intrinsic acute kidney injury. Indian J. Nephrol..

[48] Schrezenmeier E.V., Barasch J., Budde K., Westhoff T., Schmidt-Ott K.M. (2017). Biomarkers in acute kidney injury–pathophysiological basis and clinical performance. Acta Physiol..

[49] Heller F., Frischmann S., Grufcnbaum M., Zidek W., Westhoff T.H. (2011). Urinary Calprotectin and the Distinction between Prerenal and Intrinsic Acute Kidney Injury. Clin. J. Am. Soc. Nephrol..

[50] Groschet A., Morton A.J., Polyak M.M.R., Matyjaszek S., Freeman D.E. (2008). Detection of calprotectin and its correlation to the accumulation of neutrophils within equine large colon during ischaemia and reperfusion. Equine Veter-J..

[51] Schrier R.W. (2010). Early intervention in acute kidney injury. Nat. Rev. Nephrol..

[52] Ebbing J., Mathia S., Seibert F.S., Pagonas N., Bauer F., Erber B., Günzel K., Kilic E., Kempkensteffen C., Miller K. (2013). Urinary calprotectin: A new diagnostic marker in urothelial carcinoma of the bladder. World J. Urol..

[53] Seibert F.S., Rosenberger C., Mathia S., Arndt R., Arns W., Andrea H., Pagonas N., Bauer F., Zidek W., Westhoff T.H. (2017). Urinary Calprotectin Differentiates Between Prerenal and Intrinsic Acute Renal Allograft Failure. Transplantation.

[54] Hosohata K., Matsuoka H., Iwanaga K., Kumagai E. (2020). Urinary vanin-1 associated with chronic kidney disease in hypertensive patients: A pilot study. J. Clin. Hypertens..

[55] Bartucci R., Salvati A., Olinga P., Boersma Y.L. (2019). Vanin 1: Its Physiological Function and Role in Diseases. Int. J. Mol. Sci..

[56] Hosohata K., Ando H., Fujimura A. (2012). Urinary Vanin-1 As a Novel Biomarker for Early Detection of Drug-Induced Acute Kidney Injury. J. Pharmacol. Exp. Ther..

[57] Hosohata K., Matsuoka H., Kumagai E. (2021). Association of urinary vanin-1 with kidney function decline in hypertensive patients. J. Clin. Hypertens..

[58] Su Y., Lu J., Gong P., Chen X., Liang C., Zhang J. (2018). Rapamycin induces autophagy to alleviate acute kidney injury following cerebral ischemia and reperfusion via the mTORC1/ATG13/ULK1 signaling pathway. Mol. Med. Rep..

[59] von Mach T., Carlsson M.C., Straube T., Nilsson U., Leffler H., Jacob R. (2014). Ligand binding and complex formation of galectin-3 is modulated by pH variations. Biochem. J..

[60] O’Seaghdha C.M., Hwang S.-J., Ho J.E., Vasan R.S., Levy D., Fox C.S. (2013). Elevated Galectin-3 Precedes the Development of CKD. J. Am. Soc. Nephrol..

[61] Chen S.C., Kuo P.L. (2016). The role of galectin-3 in the kidneys. Int. J. Mol. Sci..

[62] Hussain M., Habib A., Najmi A. (2020). Potential predictive biomarkers for early detection of diabetic kidney disease. Int. J. Infect. Dis..

[63] Fredriksson L., Li H., Eriksson U. (2004). The PDGF family: Four gene products form five dimeric isoforms. Cytokine Growth Factor Rev..

[64] Boor P., Ostendorf T., Floege J. (2014). PDGF and the progression of renal disease. Nephrol. Dial. Transplant..

[65] Kok H.M., Falke L.L., Goldschmeding R., Nguyen T.Q. (2014). Targeting CTGF, EGF and PDGF pathways to prevent progression of kidney disease. Nat. Rev. Nephrol..

[66] Floege J., Eitner F., Alpers C.E. (2007). A New Look at Platelet-Derived Growth Factor in Renal Disease. J. Am. Soc. Nephrol..

[67] Gao L., Zhong X., Jin J., Li J., Meng X.-M. (2020). Potential targeted therapy and diagnosis based on novel insight into growth factors, receptors, and downstream effectors in acute kidney injury and acute kidney injury-chronic kidney disease progression. Signal Transduct. Target. Ther..

[68] du Cheyron D., Daubin C., Poggioli J., Ramakers M., Houillier P., Charbonneau P., Paillard M. (2003). Urinary measurement of Na+/H+ exchanger isoform 3 (NHE3) protein as new marker of tubule injury in critically ill patients with ARF. Am. J. Kidney Dis..

[69] Li X.C., Zhu D., Chen X., Zheng X., Zhao C., Zhang J., Soleimani M., Rubera I., Tauc M., Zhou X. (2019). Proximal tubule-specific deletion of the NHE3 (Na+/H+ exchanger 3) in the kidney attenuates Ang II (angiotensin II)-induced hypertension in mice. Hypertension.

[70] Ratajczyk K., Konieczny A., Czekaj A., Piotrów P., Fiutowski M., Krakowska K., Kowal P., Witkiewicz W., Marek-Bukowiec K. (2022). The Clinical Significance of Urinary Retinol-Binding Protein 4: A Review. Int. J. Environ. Res. Public Health.

[71] Garcon G., Leleu B., Zerimech F., Marez T., Haguenoer J.-M., Furon D., Shirali P. (2004). Biologic Markers of Oxidative Stress and Nephrotoxicity as Studied in Biomonitoring of Adverse Effects of Occupational Exposure to Lead and Cadmium. J. Occup. Environ. Med..

[72] D’Amico G., Bazzi C. (2003). Urinary protein and enzyme excretion as markers of tubular damage. Curr. Opin. Nephrol. Hypertens..

[73] Chen L.-S., Singh R.J. (2019). Utilities of traditional and novel biomarkers in the management of acute kidney injury. Crit. Rev. Clin. Lab. Sci..

[74] Matsui K., Kamijo-Ikemorif A., Sugaya T., Yasuda T., Kimura K. (2011). Renal Liver-Type Fatty Acid Binding Protein (L-FABP) Attenuates Acute Kidney Injury in Aristolochic Acid Nephrotoxicity. Am. J. Pathol..

[75] Kamijo A., Kimura K., Sugaya T., Yamanouchi M., Hikawa A., Hirano N., Hirata Y., Goto A., Omata M. (2004). Urinary fatty acid–binding protein as a new clinical marker of the progression of chronic renal disease. J. Lab. Clin. Med..

[76] Manabe K., Kamihata H., Motohiro M., Senoo T., Yoshida S., Iwasaka T. (2011). Urinary liver-type fatty acid-binding protein level as a predictive biomarker of contrast-induced acute kidney injury. Eur. J. Clin. Investig..

[77] Kamijo A., Kimura K., Sugaya T., Yamanouchi M., Hase H., Kaneko T., Hirata Y., Goto A., Fujita T., Omata M. (2002). Urinary free fatty acids bound to albumin aggravate tubulointerstitial damage. Kidney Int..

[78] Kamijo-Ikemori A., Sugaya T., Kimura K. (2006). Urinary fatty acid binding protein in renal disease. Clin. Chim. Acta.

[79] Assounga A.G. (2021). Beta 2 microglobulin in kidney failure: A review and an algorithm for renal replacement therapy. Saudi J. Kidney Dis. Transplant..

[80] Puthiyottil D., Priyamvada P., Kumar M.N., Chellappan A., Zachariah B., Parameswaran S. (2021). Role of Urinary Beta 2 Microglobulin and Kidney Injury Molecule-1 in Predicting Kidney Function at One Year Following Acute Kidney Injury. Int. J. Nephrol. Renov. Dis..

[81] Perbal B. (2004). CCN proteins: Multifunctional signalling regulators. Lancet.

[82] Lai C.-F., Wang J.-J., Tu Y.-C., Hsu C.-Y., Wu H.-Y., Fang C.-C., Chen Y.-M., Wu M.-S., Tsai T.-J. (2021). Associations between urinary cysteine-rich protein 61 excretion and kidney function decline in outpatients with chronic kidney disease: A prospective cohort study in Taiwan. BMJ Open.

[83] Muramatsu Y., Tsujie M., Kohda Y., Pham B., Perantoni A.O., Zhao H., Jo S.-K., Yuen P.S., Craig L., Hu X. (2002). Early detection of cysteine rich protein 61 (CYR61, CCN1) in urine following renal ischemic reperfusion injury. Kidney Int..

[84] Lai C.-F., Lin S.-L., Chiang W.-C., Chen Y.-M., Wu V.-C., Young G.-H., Ko W.-J., Kuo M.-L., Tsai T.-J., Wu K.-D. (2014). Blockade of cysteine-rich protein 61 attenuates renal inflammation and fibrosis after ischemic kidney injury. Am. J. Physiol. Physiol..

[85] Schnaper Schnaper H.W., Jandeska S., Runyan C.E., Hubchak S.C., Basu R.K., Curley J.F., Smith R.D., Hayashida T. (2009). TGF-beta signal transduction in chronic kidney disease. Front. Biosci. (Landmark Ed.).

[86] Harskamp L.R., Gansevoort R.T., Van Goor H., Meijer E. (2016). The epidermal growth factor receptor pathway in chronic kidney diseases. Nat. Rev. Nephrol..

[87] Samarakoon R., Dobberfuhl A.D., Cooley C., Overstreet J.M., Patel S., Goldschmeding R., Meldrum K.K., Higgins P.J. (2013). Induction of renal fibrotic genes by TGF-β1 requires EGFR activation, p53 and reactive oxygen species. Cell. Signal..

[88] Kilari S., Yang B., Sharma A., McCall D.L., Misra S. (2018). Increased transforming growth factor beta (TGF-β) and pSMAD3 signaling in a Murine Model for Contrast Induced Kidney Injury. Sci. Rep..

[89] Lai J.Y., Luo J., O’Connor C., Jing X., Nair V., Ju W., Randolph A., Ben-Dov I.Z., Matar R.N., Briskin D. (2015). MicroRNA-21 in glomerular injury. J. Am. Soc. Nephrol..

[90] Roberts A.B. (1998). Molecular and cell biology of TGF-β. Miner. Electrolyte Metab..

[91] Chung A.C.K., Lan H.Y. (2013). Molecular Mechanisms of TGF-β Signaling in Renal Fibrosis. Curr. Pathobiol. Rep..

[92] Wang J.-N., Yang Q., Yang C., Cai Y.-T., Xing T., Gao L., Wang F., Chen X., Liu X.-Q., He X.-Y. (2020). Smad3 promotes AKI sensitivity in diabetic mice via interaction with p53 and induction of NOX4-dependent ROS production. Redox Biol..

[93] Morita A., Numata Y., Kosugi Y., Noto A., Takeuchi N., Uchida K. (1998). Stabilities of N-acetyl-β-d-glucosaminidase (NAG) isoenzymes in urine: Advantage of NAG isoenzyme B measurement in clinical applications. Clin. Chim. Acta.

[94] Hong J.D., Lim I.S. (2012). Correlation between glomerular filtration rate and urinary N acetyl-beta-D glucosaminidase in children with persistent proteinuria in chronic glomerular disease. Korean J. Pediatr..

[95] Bondiou M.T., Bourbouze R., Bernard M., Percheron F., Perez-Gonzalez N., Cabezas J.A. (1985). Inhibition of A and B N-acetyl-β-d-glucosaminidase urinary isoenzymes by urea. Clin. Chim. Acta.

[96] Kim S.R., Lee Y.H., Lee S.G., Kang E.S., Cha B.S., Kim J.H., Lee B.W. (2016). Urinary N-acetyl-β-D-glucosaminidase, an early marker of diabetic kidney disease, might reflect glucose excursion in patients with type 2 diabetes. Medicine.

[97] Kadokura T., Saito M., Utsuno A., Kazuta K., Yoshida S., Kawasaki S., Nagase I., Kageyama S. (2011). Ipragliflozin (ASP1941), a selective sodium-dependent glucose cotransporter 2 inhibitor, safely stimulates urinary glucose excretion without inducing hypoglycemia in healthy Japanese subjects. Diabetol. Int..

[98] Hart S.G. (2005). Assessment of renal injury in vivo. J. Pharmacol. Toxicol. Methods.

[99] Hartner A., Porst M., Gauer S., Pröls F., Veelken R., Hilgers K.F. (2001). Glomerular osteopontin expression and macrophage infiltration in glomerulosclerosis of DOCA–salt rats. Am. J. Kidney Dis..

[100] Varalakshmi B., Kiranmyai V.S., Aparna B., Ram R., Rao P.V.L.N.S., Kumar V.S. (2020). Plasma osteopontin levels in patients with acute kidney injury requiring dialysis: A study in a tertiary care institute in South India. Int. Urol. Nephrol..

[101] Magistroni R., D’Agati V.D., Appel G.B., Kiryluk K. (2015). New developments in the genetics, pathogenesis, and therapy of IgA nephropathy. Kidney Int..

[102] Cattran D.C., Brenchley P.E. (2017). Membranous nephropathy: Integrating basic science into improved clinical management. Kidney Int..

[103] Mezzano S.A., Barría M., Droguett M.A., Burgos M.E., Ardiles L.G., Flores C., Egido J. (2001). Tubular NF-κB and AP-1 activation in human proteinuric renal disease. Kidney Int..

[104] Perkins N.D. (2007). Integrating cell-signalling pathways with NF-κB and IKK function. Nat. Rev. Mol. Cell Biol..

[105] Yan X., Sano M., Lu L., Wang W., Zhang Q., Zhang R., Wang L., Chen Q., Fukuda K., Shen W. (2010). Plasma concentrations of osteopontin, but not thrombin-cleaved osteopontin, are associated with the presence and severity of nephropathy and coronary artery disease in patients with type 2 diabetes mellitus. Cardiovasc. Diabetol..

[106] Zhou J., Chen H., Fan Y. (2017). Systematic analysis of the expression profile of non-coding RNAs involved in ischemia/reperfusion-induced acute kidney injury in mice using RNA sequencing. Oncotarget.

[107] Guo J., Guan Q., Liu X., Wang H., Gleave M.E., Nguan C.Y.C., Du C. (2016). Relationship of clusterin with renal inflammation and fibrosis after the recovery phase of ischemia-reperfusion injury. BMC Nephrol..

[108] Davis J.W., Goodsaid F.M., Bral C.M., Obert L.A., Mandakas G., Garner I.I.C.E., Collins N.D., Smith R.J., Rosenblum I.Y. (2004). Quantitative gene expression analysis in a nonhuman primate model of antibiotic-induced nephrotoxicity. Toxicol. Appl. Pharmacol..

[109] Park S., Mathis K.W., Lee I.K. (2013). The physiological roles of apolipoprotein J/clusterin in metabolic and cardiovascular diseases. Rev. Endocr. Metab. Disord..

[110] Wadey R.M., Pinches M.G., Jones H.B., Riccardi D., Price S. (2013). Tissue Expression and Correlation of a Panel of Urinary Biomarkers Following Cisplatin-induced Kidney Injury. Toxicol. Pathol..

[111] Jones S.A., Horiuchi S., Topley N., Yamamoto N., Fuller G.M. (2000). The soluble interleukin 6 receptor: Mechanisms of production and implications in disease. FASEB J..

[112] Shalaby M., Waage A., Espevik T. (1989). Cytokine regulation of interleukin 6 production by human endothelial cells. Cell. Immunol..

[113] Nechemia-Arbely Y., Barkan D., Pizov G., Shriki A., Rose-John S., Galun E., Axelrod J.H. (2008). IL-6/IL-6R axis plays a critical role in acute kidney injury. J. Am. Soc. Nephrol..

[114] Taddei M.L., Giannoni E., Fiaschi T., Chiarugi P. (2011). Anoikis: An emerging hallmark in health and diseases. J. Pathol..

[115] Meng X.-M., Nikolic-Paterson D.J., Lan H.Y. (2016). TGF-β: The master regulator of fibrosis. Nat. Rev. Nephrol..

[116] Devaux C.A., Mezouar S., Mege J.-L. (2019). The E-Cadherin Cleavage Associated to Pathogenic Bacteria Infections Can Favor Bacterial Invasion and Transmigration, Dysregulation of the Immune Response and Cancer Induction in Humans. Front. Microbiol..

[117] Koziolek M., Mueller G.A., Dihazi G.H., Jung K., Altubar C., Wallbach M., Markovic I., Raddatz D., Jahn O., Karaköse H. (2020). Urine E-cadherin: A Marker for Early Detection of Kidney Injury in Diabetic Patients. J. Clin. Med..

[118] Parmentier M., Ghysens M., Rypens F., Lawson D., Pasteels J., Pochet R. (1987). Calbindin in vertebrate classes: Immunohistochemical localization and Western blot analysis. Gen. Comp. Endocrinol..

[119] George B., Szilagyi J.T., Joy M.S., Aleksunes L.M. (2022). Regulation of renal calbindin expression during cisplatin-induced kidney injury. J. Biochem. Mol. Toxicol..

[120] Eltounali S.A., Moodley J., Naicker T. (2017). Role of kidney biomarkers [Kidney injury molecule-1, Calbindin, Interleukin-18 and Monocyte chemoattractant protein-1] in HIV associated pre-eclampsia. Hypertens. Pregnancy.

[121] Maiwall R., Kumar A., Bhardwaj A., Kumar G., Bhadoria A.S., Sarin S.K. (2017). Cystatin C predicts acute kidney injury and mortality in cirrhotics: A prospective cohort study. Liver Int..

[122] Zhang Z., Lu B., Sheng X., Jin N. (2011). Cystatin C in Prediction of Acute Kidney Injury: A Systemic Review and Meta-analysis. Am. J. Kidney Dis..

[123] Deng Y., Wang L., Hou Y., Ma J., Chi R., Ye H., Zhai Y., Zhang D., Gao L., Hu L. (2019). The influence of glycemic status on the performance of cystatin C for acute kidney injury detection in the critically ill. Ren. Fail..

[124] Yang Q.-H., Liu D.-W., Long Y., Liu H.-Z., Chai W.-Z., Wang X.-T. (2009). Acute renal failure during sepsis: Potential role of cell cycle regulation. J. Infect..

[125] Godi I., De Rosa S., Martino F., Bazzano S., Martin M., Boni E., Carta M.R., Diaz C.T., Mari G., Lorenzin A. (2020). Urinary [TIMP-2] × [IGFBP7] and serum procalcitonin to predict and assess the risk for short-term outcomes in septic and non-septic critically ill patients. Ann. Intensiv. Care.

[126] Seo D.-W., Li H., Qu C.-K., Oh J., Kim Y.-S., Diaz T., Wei B., Han J.-W., Stetler-Stevenson W.G. (2006). Shp-1 Mediates the Antiproliferative Activity of Tissue Inhibitor of Metalloproteinase-2 in Human Microvascular Endothelial Cells. J. Biol. Chem..

[127] Gunnerson K.J., Shaw A.D., Chawla L., Bihorac A., Al-Khafaji A., Kashani K., Lissauer M., Shi J., Walker M.G., Kellum J.A. (2016). TIMP2•IGFBP7 biomarker panel accurately predicts acute kidney injury in high-risk surgical patients. J. Trauma Inj. Infect. Crit. Care.

[128] Maizel J., Daubin D., Van Vong L., Titeca-Beauport D., Wetzstein M., Kontar L., Slama M., Klouche K., Vinsonneau C. (2019). Urinary TIMP2 and IGFBP7 Identifies High Risk Patients of Short-Term Progression from Mild and Moderate to Severe Acute Kidney Injury during Septic Shock: A Prospective Cohort Study. Dis. Markers.

